# How Are Mechanical, Physiological, and Perceptual Variables Affected by the Rest Interval Between Sets During a Flywheel Resistance Session?

**DOI:** 10.3389/fphys.2020.00663

**Published:** 2020-06-16

**Authors:** Rafael Sabido, Jose Luis Hernández-Davó, Laia Capdepon, Julio Tous-Fajardo

**Affiliations:** ^1^Sport Science Department, Miguel Hernández University, Elche, Spain; ^2^Faculty of Health Sciences, Universidad Isabel I de Castilla, Burgos, Spain; ^3^Sports Performance Laboratory, Instituto Nacional de Educación Física de Cataluña (INEFC), Barcelona, Spain

**Keywords:** power, strength, isoinertial, performance, fatigue

## Abstract

The use of flywheel devices has increased in popularity during the last few years. Flywheel training is an attractive alternative to traditional resistance exercise because it allows for the loading stimulus to be manipulated. Some of the benefits associated with flywheel training include increases in muscle hypertrophy, muscular strength and reductions in injury risk. Nevertheless, there is a lack of research about how basic training variables [i.e., rest intervals (RI) between sets], or variables associated with manipulation of the loading stimulus (i.e., different inertial loads), influence the acute responses during a flywheel session. Thus, the aim of this study was to assess the influence of three different RI between sets (1, 2, or 3 min), during a flywheel squat session with two different inertial loads: light (0.025 kg⋅m^2^) and high (0.075 kg⋅m^2^). Twenty-three participants performed six exercise sessions (two inertial loads × three RI) consisting of four sets of 11 repetitions. Concentric and eccentric power, lactate concentration ([La]) and ratings of perceived exertion (RPE) were measured during the exercise session, and delayed onset muscular soreness (DOMS) were recorded 24 h post-exercise. Both concentric (9.1 and 22.1% at light and high load respectively; *p* = 0.022 and 0.005) and eccentric peak power (17.5% at high load; *p* = 0.02) decreased across sets when the 1 min RI was used. Concentric peak power was decreased (11.1%, *p* = 0.041) from the 2 min RI only with the high inertial load. RPE was higher during the 1 min compared with the 3 min RI protocol when using the high inertial load (*p* = 0.028). [La] was higher when using the 2 min RI compared with the 3 min RI at light load (*p* = 0.03). In conclusion, during flywheel training sessions, a short RI (1 min) was insufficient to maintain power output across all four sets and was linked to greater perceptual variables. A 2 min RI allowed for power to be maintained, but only when training with light inertial loads. Based on these results, coaches should consider prescribing 3 min RI’s when performing flywheel squat exercises regardless the inertial load. In contrast, when using 2 min RI’s the inertial load should be light.

## Introduction

Strength training is considered crucial for the progressive adaptation in muscular strength and performance (American College of Sports Medicine [ACSM], 2009). In order to create an effective and efficient strength training program, it is essential to manage variables such as intensity, volume, and rest interval (RI) between sets (de Salles et al., 2009). The selection of a given RI is usually based on the aim of the training session (Willardson, 2006). Long (e.g., 2–5 min) RI’s are commonly implemented when training for strength and power adaptations, while shorter RIs are recommended for muscular hypertrophy (30–120 s) and endurance (30–60 s) (Willardson, 2006). Specifically, when training for power adaptations, some authors have recommended long RIs between sets (>3 min) (Abdessemed et al., 1999; Pincivero and Campy, 2004), based on the fact that power output is dependent on the phosphagen energy system.

The manipulation of the RI between sets affects the acute responses to a strength training session and has an influence on the chronic neuromuscular and endocrine adaptations after a training period. During resistance training sessions, muscular performance is primarily dependent on anaerobic energy metabolism (phosphagen system). Greater energy supply by the glycolytic system is required when RI’s are too short for full recovery of adenosin triphosphate (ATP) and phosphocreatine (PCr) to occur. Therefore, hydrogen ions might accumulate, resulting in a decrease in intramuscular pH. This effect might negatively influence muscular performance by reducing force and/or power production (Pincivero and Campy, 2004; de Salles et al., 2009). In addition, the duration of the RI between sets might have an impact on the acute endocrine responses. Rahimi et al. (2010) showed that a short (60 s) RI led to greater growth hormone concentrations, while longer (120 s) RI entailed higher testosterone responses. The detrimental effects in acute neuromuscular and hormonal responses resulting from RI’s that are too short may impact chronic adaptations, including strength gains, hypertrophic effects, and sensitivity of the muscles receptors to circulating hormones (Kraemer and Ratamess, 2005; de Salles et al., 2009).

Ellington (2001) previously showed that the level of phosphagens present in the muscle fibers is related to maximum potential rates of ATP turnover and oxidative capacity, which is correlated with power output. Some authors have suggested that short RI’s prevent the ATP and PCR stores from being fully replenished, which can lead to increases in lactate concentration as well as decreases in power output (Ratamess et al., 2007). In contrast, other authors have reported similar decrements in power output within a training session when using either short (1 min) or long (4 min) RI’s (Nibali et al., 2013). These discrepancies may be attributed to differences in the exercise analyzed (bench press vs. squat), the intensity used (0 vs. 70% of 1 repetition maximum), or the relative strength (1 RM/body mass) of the participants (Abdessemed et al., 1999; Nibali et al., 2013; Hernández-Davó et al., 2017).

Although the influence of varying durations of RI’s on both acute responses and chronic adaptations to strength training when using constant gravitational loads has been widely studied (Willardson, 2006; de Salles et al., 2009), little is known about the effects of different RI’s taken between sets of exercise during flywheel resistance training sessions. The flywheel device was created to allow the user to manipulate the traditional constant gravitational load. Specifically, by using the inertia of a rotating flywheel, the device allows the user to produce greater force/power during the eccentric than the concentric phase of the movement, leading to the so-called, eccentric-overload training (Martínez-Aranda and Fernández-Gonzalo, 2017). The use of eccentric-overload training has increased in popularity (Gual et al., 2014; Tous-Fajardo et al., 2016; Sabido et al., 2017), likely due to its efficacy in injury prevention and rehabilitation (Romero-Rodríguez et al., 2011), muscular hypertrophy (Norrbrand et al., 2008) and performance optimization (Tous-Fajardo et al., 2016; Sabido et al., 2017).

In spite of the growing use of eccentric-overload training, there is a scarcity of research about the influence of basic training variables such as volume, intensity and RI’s between sets on either acute or chronic responses after using flywheel training. Only two studies have reported that the training intensity (i.e., inertial load) affects both concentric and eccentric power output, as well as its ratio (Martínez-Aranda and Fernández-Gonzalo, 2017; Sabido et al., 2017). Specifically, light inertial loads allow for greater concentric and eccentric power to be produced, while eccentric overload (eccentric/concentric ratio) is maximized when using high loads. Studies utilizing flywheel training have most commonly implemented 2 min RI’s (Gual et al., 2016; Tous-Fajardo et al., 2016; Sabido et al., 2017). However, other studies using flywheel training have implemented 1 min RI’s (Askling et al., 2003) and 3 min RI’s (Maroto-Izquierdo et al., 2017). Despite this variety in RI used during flywheel training, to the best of authors’ knowledge, no studies have analyzed the effect of different RI’s on either acute power responses or chronic neuromuscular adaptations. Therefore, the main objective of this study was to investigate the influence of different RI’s between sets (i.e., 1, 2, and 3 min) on power output production during consecutive sets of the flywheel squat exercise using two different inertial loads (light and high). As a secondary objective, lactate concentration, ratings of perceived exertion (RPE) and delayed onset muscular soreness (DOMS) was measured in order to determine whether the duration of the RI may have an effect on these variables. We hypothesized that independent of inertial load, only a 3 min RI would allow for power output to be maintained across the sets. Secondarily, we hypothesized that the shorter RI would result in greater RPE and lactate concentrations.

## Materials and Methods

### Participants

Twenty-three male handball players (age: 24.4 ± 4.3 years; height: 1.83 ± 0.07 m; body mass: 80.7 ± 6.3 kg) voluntarily participated in the study. Sample size estimation based on power output variables (100 W difference; *SD* = 250 W; 80% power, *p* < 0.05), revealed that a sample size of 17 participants was needed to find significant differences. All the participants were carefully informed about the potential risk of the testing sessions and signed written informed consent approved by the Ethics Committee of the University in accordance with the Declaration of Helsinki before participation. Throughout the investigation, participants were requested to maintain their regular diets and normal hydration state, not to take any nutritional supplementation or anti-inflammatory medications, and to refrain from caffeine intake in the 3 h before each testing session. Vigorous exercise was not allowed at least 24 h before the testing sessions.

### Exercise Protocol

To assess the influence of different RI’s between sets on mechanical, physiological, and perceptual variables during a flywheel squat session, each participant completed 6 testing sessions: one for each inertial load [light (0.025 kg⋅m^2^) and high (0.075 kg⋅m^2^)] and each rest interval between sets (1, 2, or 3 min). Each session consisted of four sets of 11 repetitions of the flywheel half-squat exercise. During the sessions, concentric and eccentric power, lactate concentration (1 min post), and RPE (5 min post) were measured. In addition, DOMS 24 h post-session was also registered.

Participants completed eight laboratory sessions in total. Before testing sessions, all participants attended two familiarization sessions. During the familiarization sessions, participants performed four sets of 10 repetitions (two sets with each inertial load). Previous research has shown that two familiarization sessions are required to find consistent and reliable data from the flywheel squat exercise (Sabido et al., 2017). Participants then completed six testing sessions, each separated by 1 week. To avoid experimental variability the same two researches carried out the testing sessions and all participants were scheduled at the same hour during the six testing sessions. The testing sessions differed in the RI used between sets (i.e., 1, 2, or 3 min) and the inertial load used (i.e., light or high). The order of the testing sessions was counter balanced among all the participants. The first three repetitions of each set were used to initiate the movement and excluded from data analysis. Then, participants performed eight maximal effort repetitions. To standardize the participants’ range of motion, a tape was placed between two posts of a rack located just behind the participants to ensure that the participants attained a knee joint angle of 90°. Thus, the participants performed the squat movement from the lower (90°) position to the full extension of the knees (180°). The participants were instructed to perform the concentric phase as fast as possible while delaying the braking action to the last third of the eccentric phase. Loud verbal encouragement was given to the participants during all testing sessions.

#### Mechanical Data

During each repetition, both concentric and eccentric power were recorded by means of an optical receiver (SmartCoach, Europe AB, Stockholm, Sweden) coupled to the flywheel device. The information was then processed using specialized software (SmartCoach Power Encoder, Europe AB, Stockholm, Sweden). The variables used for data analysis were peak concentric (PPconc) and peak eccentric power (PPecc) and the eccentric/concentric ratio (i.e., peak eccentric power/peak concentric power; Ecc/Conc Ratio).

#### Lactate Concentration

Lactate concentration was determined from capillary blood samples (0.5 μL) drawn from the earlobe (Tanner et al., 2010). After removing the first blood drop, the second drop was collected with a reactive test strip and analyzed with a portable device (Lactate Scout; Senselab, Leipzig, Germany), with an accuracy of 0.1 mmol. Samples were taken 1 min after each protocol and analyzed at this time point by the portable lactate analyzer.

#### Rating of Perceived Exertion (RPE) and Delayed Onset Muscular Soreness (DOMS)

The Borg category scale (CR-10) was used to determine the participants’ rating of perceived exertion during exercise. The CR-10 scale was defined by the following anchor points: “rest” (0) and “maximal” (10). Participants were asked, *“How hard do you feel the exercise was?”* 5 min after the last set of each session. DOMS was reported by the participants 24 h after each session. Before their habitual on-court practice, and after a standardized 6–8 min general warm-up consisting of low intensity jogging, multilateral displacements and dynamic stretching, participants were asked, *“How painful do your quadriceps muscles feel?”* giving their subjective feeling on a 0–10 scale (0 = no pain; 10 = a lot of pain) (Ojala and Hakkinen, 2013). All participants reported no DOMS before all testing sessions.

### Statistical Analysis

Data are presented as mean ± SD. All statistical analyses were carried out using the SPSS 23.0 (SPSS Inc., Chicago, IL, United States). A two-way repeated measures ANOVA (rest interval x inertial load) was used to evaluate lactate concentration and RPE, and DOMS data, whereas another two-way repeated measures ANOVA (rest interval x set) was used to evaluate interset mechanical power data in both inertial load protocols. When significant interactions were found, a Bonferroni *post hoc* was used for pairwise comparisons. Statistical significance was set at *p* < 0.05. In addition, the Cohen’s *d* effect size (ES) was used to evaluate the magnitude of the differences and interpreted as trivial (<0.25), small (0.25–0.50), moderate (0.50–0.80), and large (>0.80) (Rhea, 2004). For mechanical variables, the data analysis was performed using the mean of the eight repetitions for each set.

## Results

**FIGURE 1:**
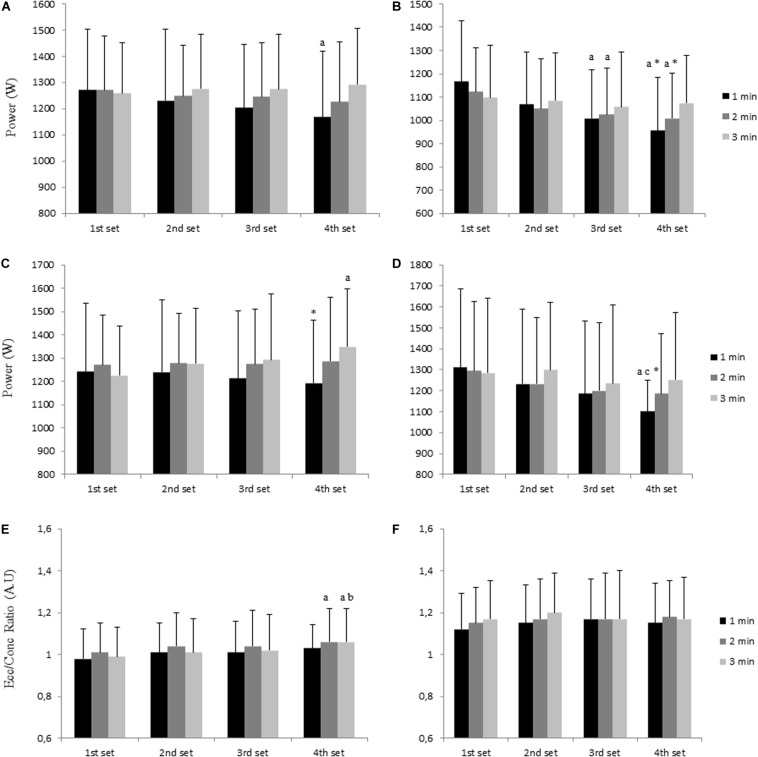
Mechanical data per set for each RI condition for the variables and flywheel resistance of PPconc (**A:** 0.025 kg⋅m^−1^; **B:** 0.075 kg⋅m^−1^), PPecc (**C:** 0.025 kg⋅m^−1^; **D:** 0.075 kg⋅m^−1^), and Ecc/Conc Ratio (**E:** 0.025 kg⋅m^−1^; **F:** 0.075 kg⋅m^−1^). a, significant difference with 1st set; b, significant difference with 2nd set; c, significant difference with 3rd set; *, significant difference with 3 min RI.

Concentric (PPconc), eccentric (PPecc) peak power, and eccentric to concentric power ratio (Ecc/Conc Ratio) across each set, RPE and lactate concentration [La^–^] after the session, and muscular soreness (DOMS) at 24 h for the three different rest interval durations are presented in Table 1 for the light load and in Table 2 for the high load. In addition, the comparisons of mechanical data between the different RI protocols are shown in Figure 1.

**TABLE 1:** Perceptual variables, physiological variables, and mechanical data over the sets with the light inertial load by rest interval.

	PP_conc_	PP_ecc_	Ecc/Conc ratio	RPE	[La^–^]	DOMS 24 h
**1 min RI**						
1st set	1273 ± 237	1230 ± 295	0.97 ± 0.14	6.9 ± 1.0	4.6 ± 1.7	7.7 ± 1.2
2nd set	1243 ± 271	1236 ± 310	1.00 ± 0.14			
3rd set	1196 ± 240	1210 ± 282	1.02 ± 0.15			
4th set	1167 ± 252^ab^	1192 ± 271	1.03 ± 0.11			
**2 min RI**						
1st set	1270 ± 210	1262 ± 212	1.00 ± 0.14^d^	6.0 ± 1.5	5.5 ± 1.5	7.3 ± 1.5
2nd set	1263 ± 197	1294 ± 220	1.04 ± 0.16			
3rd set	1236 ± 208	1265 ± 240	1.03 ± 0.17			
4th set	1224 ± 228	1302 ± 271	1.08 ± 0.17			
**3 min RI**						
1st set	1266 ± 188	1231 ± 218^d^	0.98 ± 0.14^d^	6.2 ± 1.9	4.3 ± 1.6^#^	7.2 ± 1.3
2nd set	1284 ± 210	1274 ± 245	1.00 ± 0.16^d^			
3rd set	1269 ± 207	1294 ± 277	1.03 ± 0.17			
4th set	1292 ± 217	1364 ± 255*	1.07 ± 0.18			

*^a^Significantly lower than 1st set. ^b^Significantly lower than 2nd set. ^d^Significantly lower than 4th set. ^∗^Significant difference with 1 min RI. ^#^Significant difference with 2 min RI.*

**TABLE 2:** Perceptual variables, physiological variables, and mechanical data over the sets with the high inertial load by rest interval.

	PP_conc_	PP_ecc_	Ecc/Conc ratio	RPE	[La^–^]	DOMS 24 h
**1 min RI**						
1st set	1143 ± 269	1298 ± 367	1.14 ± 0.18	7.4 ± 1.3	5.2 ± 1.8	7.5 ± 1.0
2nd set	1053 ± 226^a^	1226 ± 345	1.16 ± 0.18			
3rd set	987 ± 205^ab^	1188 ± 337	1.20 ± 0.19			
4th set	936 ± 223^ab^	1105 ± 346^ac^	1.17 ± 0.20			
**2 min RI**						
1st set	1112 ± 191	1275 ± 336	1.14 ± 0.18	7.3 ± 1.4	4.5 ± 1.9	7.7 ± 0.9
2nd set	1043 ± 218	1211 ± 315	1.16 ± 0.19			
3rd set	1013 ± 204^a^	1207 ± 336	1.19 ± 0.22			
4th set	989 ± 197^a^	1169 ± 287	1.19 ± 0.17			
**3 min RI**						
1st set	1106 ± 214	1267 ± 341	1.15 ± 0.18	6.5 ± 1.5*	4.5 ± 1.8	7.9 ± 0.9
2nd set	1085 ± 197	1283 ± 308	1.19 ± 0.19			
3rd set	1055 ± 225*	1229 ± 352	1.16 ± 0.22			
4th set	1061 ± 206*^#^	1241 ± 316*	1.17 ± 0.20			

*^a^Significantly lower than 1st set. ^b^Significantly lower than 2nd set. ^c^Significantly lower than 3rd set. ^d^Significantly lower than 4th set. ^∗^Significant difference with 1 min RI. ^#^Significant difference with 2 min RI.*

Over the four sets, PPconc and PPecc at light load was maintained under both 2 and 3 min RI conditions (*p* > 0.05). Although PPecc was also maintained across all four sets during the 1 min RI, PPconc decreased across sets (*p* = 0.030) showing the 4th set significantly lower values (*p* = 0.022), and the 3rd set a trend for lower values (*p* = 0.067). At the high load, PPconc was decreased across sets (*p* < 0.001). Specifically, PPconc was reduced in the 3rd (*p* = 0.041) and 4th (*p* = 0.047) set for the 2 min RI condition, and in the 2nd (*p* = 0.008), 3rd (*p* = 0.004), and 4th (*p* = 0.005) set for the 1 min RI condition. However, PPconc was not reduced across sets for the 3 min RI condition. PPecc was maintained across sets under both 2 and 3 min RI conditions, except the 4th set under the 3 min RI condition, which showed significant greater PPecc values (*p* = 0.041) than the 1st set. Contrarily, under the 1 min RI condition, a significant decrease in PPecc was found in the 4th set compared with the 3rd (*p* = 0.034) and the 1st set (*p* = 0.020). In addition, when using the high load, significant interactions were found. Specifically, PPconc in the 3rd set was lower for the 1 compared with the 3 min RI condition (*p* = 0.014). The 4th set also showed lower PPconc values during both the 1 min RI (*p* = 0.010) and the 2 min RI (*p* = 0.008) compared with the 3 min RI condition. PPecc also showed significant interactions at high load, showing the 1 min RI lower values during the 4th set (*p* = 0.010) compared with the 3 min RI condition. Finally, a rest x set interaction was found for PPecc at light load showing the 1 min RI lower PPecc values in the 4th set than the 3 min RI condition (*p* = 0.020).

For Ecc/Conc Ratio, at light load, a greater Ecc/Conc ratio was found in the 4th set compared with the 1st (*p* = 0.014) under the 2 min RI condition. In addition, under the 3 min RI condition, higher Ecc/Conc ratio values were found in the 4th set compared with the 1st (*p* = 0.013) and 2nd (*p* = 0.025) set. No differences were found for Ecc/Conc ratio across sets at high load.

Regarding perceptual variables, at light load, [La] was significantly higher under the 2 min RI protocol compared with the 3 min RI (*p* = 0.030). In addition, RPE under the 2 min RI protocol was significantly higher at light load compared with that at high load. In addition, at high load RPE was significantly higher under the 1 min RI than under the 3 min RI condition (*p* = 0.028). No differences were found for DOMS values.

## Discussion

The aims of this study were to test the influence of different RI’s on mechanical, physiological and perceptual variables during the flywheel squat exercise at two different inertial loads. The main finding was that the 1 min RI protocol entailed significant concentric (at light and high load) and eccentric (at high load) power output decrements, as well as increases in RPE compared with the 3 min RI protocol at high load. The 2 min RI protocol led to concentric power output decrements at high load, and elevated lactate concentrations compared to the 3 min RI at light load. Therefore, the results of the present study demonstrated that the 3 min RI is preferred as it allowed for power output to be maintained across sets, while minimizing rating of perceived exertion and lactate concentration.

With the light load, the power output across sets showed that only the shorter RI protocol (1 min) led to decreases in concentric peak power commencing from the 3rd set. In contrast, Nibali et al. (2013) suggested that 1 min RI allowed power output to be maintained over the sets of the squat exercise. It seems that, compared to traditional resistance exercises, the higher neuromuscular demands elicited by flywheel exercises (Norrbrand et al., 2010), cause the need for longer RI to be used to maintain power output during training. Several authors have previously suggested that short RI’s permit power output maintenance in the squat (Paulo et al., 2012) and squat jump exercise (Nibali et al., 2013). It should be noted, however, that both above-mentioned works used gravitational loads, highlighting the superior requirements of flywheel exercises (e.g., greater muscle activation and force production) (Tesch et al., 2017). In addition, differences in the training volume also contribute to the contrasting results. While Paulo et al. (2012) and Nibali et al. (2013) performed sets ranging from 3 to 6 repetitions, in the present study participants performed sets of 11 repetitions. Since training sessions aiming to develop power usually involve the performance of more than six repetitions per set, our data may be more practically relevant than the aforementioned studies. Based on the results found in our study, it seems that when using light inertial loads, RI’s of 2 min allows for high power output to be maintained. In fact, when comparing the 4th set with the 1st set of the training session, both 2 and 3 min RI showed slight increases in eccentric peak power, suggesting a potential post-activation potentiation (PAP) effect. This PAP effect is defined as an increase in muscle performance after a maximal or submaximal contraction, including greater rate of force developments and power production (Tillin and Bishop, 2009). Specifically, increases in power performance after a conditioning protocol based on the flywheel squat exercise has been previously reported (Cuenca-Fernández et al., 2015). There are several mechanisms underlying the PAP phenomenon, as increases in intramuscular calcium sensitivity, increases in the number of neurotransmitters and their effectiveness, and changes in the pennation angle of the muscle.

When comparing power output between different RI protocols, both concentric and eccentric peak power were unaffected by the 4th set when the participants took the 3 min RI as opposed to the 1 min RI protocol (see Figure 1). Importantly, this effect was observed independently of the inertial load used. This fact may have an important implication for training prescription, as the ability to sustain total work performed during training have been linked to greater increases in muscular strength (Robinson et al., 1995; Pincivero and Campy, 2004). Nevertheless, it should be noted that the percentage of power loss differed considerably depending on the inertial load used. For example, peak power during the 1 min RI decreased by 9.1% and by 3.2% (for concentric and eccentric phases respectively) with the light load, but by 22.1% and by 17.5% with the high load. In the same line, when using the 2 min RI protocol, PPconc decreased by 3.6% and by 11.1% (at light and high load respectively). This is of significant practical use, as the management of performance loss during training sessions influence training adaptations (Pareja-Blanco et al., 2016). Thus, based on our findings, a 1 min RI could be prescribed when using light inertial loads, but this might not be appropriate when using a high inertial load. For example, if a 10% of power loss is allowed during a session, the athlete could use RI of 1 min when using a light load. However, when using a high load (e.g., 0.075 kg⋅m^2^) 1 min RI would lead to greater power decrements (17–22% in the present study).

Perceptual variables such as RPE and DOMS have been accepted as reliable measures to gauge sessions intensity (Robertson et al., 2000; Ojala and Hakkinen, 2013). In the present study, RPE values differed significantly between RI protocols. Specifically, the 1 min RI protocol resulted in higher RPE values than the 3 min RI with the high load. These results are in line with previous research showing increases in RPE scores when short (e.g., 1 min) RI are used during strength training sessions (Scudese et al., 2015; Hernández-Davó et al., 2016). Regarding DOMS, participants reported high values (7.2–7.9 in a 0–10 scale), but no significant differences between RI protocols were found 24 h after training. These high values of soreness may reflect muscle damage, which is usually found after flywheel sessions (Fernández-Gonzalo et al., 2014). It would have been interesting to collect data of DOMS 48 h after the training session, as previous studies with these devices showed the peak of creatine kinase concentration takes place 48 h after training (Fernández-Gonzalo et al., 2014). Lactate concentration was always slightly lower when using the 3 min RI protocol, although these lower values were only significant compared to the 2 min RI protocol at light load. Thus, although performance impairments (e.g., peak force losses) have been linked to disturbances in ions (H^+^) concentrations (de Salles et al., 2009), it seems that, in the present study, this was not the direct source of power output decreases. It could be hypothesized that the nature of the training session (e.g., power session, with not to failure training) and the low fatigue index shown during the sessions (<23% of power loss) would have avoided the appearance of high lactate concentrations. This is supported by the results of Abdessemed et al. (1999), who showed that the greater differences in lactate concentrations when using different RI protocols (e.g., 1, 3, and 5 min) appeared from the 4th to the 10th set performed until repetition failure.

The current study presents some limitations. Specifically, regarding muscular soreness, additional measures at longer time points (e.g., 48–72 h) could have provided important information about the time-course recovery of muscle damage after flywheel sessions. In addition, a greater training volume (e.g., additional sets) would have been interesting to assess potential differences in lactate concentrations. It would be also interesting to assess the mechanical, perceptual and physiological responses under different RI protocols when participants use the inertial load that elicit the peak power value. Finally, the present study only utilized male trained athletes (handball players), with previous experience in the flywheel squat exercise. Therefore, the results may not translate neither to untrained individuals, nor to a female population.

## Conclusion

In conclusion, the results of the present study suggest that 1 min of rest interval between sets are insufficient to sustain power output values during a flywheel squat training session. When using light inertial loads, 2 min of rest interval can be enough to maintain training intensity but could be insufficient when training with high inertial loads. These results are of significant practical use, as are the first to show that rest interval between sets during flywheel training should be selected based on (a) the target percentage of power loss, (b) the inertial load used, and (c) the desired perceptual and physiological responses.

## Data Availability Statement

The raw data supporting the conclusions of this article will be made available by the authors, without undue reservation, to any qualified researcher.

## Ethics Statement

The studies involving human participants were reviewed and approved by the Miguel Hernández University Ethics Committee. The patients/participants provided their written informed consent to participate in this study.

## Author Contributions

JH-D and RS contributed to the conception and design of study. RS and LC contributed to the acquisition of data. LC and JH-D contributed to the drafting the manuscript. RS and JT-F contributed to the revising the manuscript critically for important intellectual content. All authors analysis and interpretation of data and approval of the version of the manuscript to be published.

## Conflict of Interest

The authors declare that the research was conducted in the absence of any commercial or financial relationships that could be construed as a potential conflict of interest.
